# Conflicting interpretations and FDA reputation: the case of post-market surveillance of breast implants

**DOI:** 10.3389/fmed.2024.1475992

**Published:** 2024-11-06

**Authors:** Moshe Maor, Yehuda Shoenfeld

**Affiliations:** ^1^Lauder School of Government, Diplomacy and Strategy, Reichman University, Herzliya, Israel; ^2^Zabludowicz Center for Autoimmune Diseases, Sheba Medical Center, Tel-Hashomer, Israel; ^3^Dina Recanati School of Medicine, Reichman University, Herzelia, Israel

**Keywords:** regulatory error, voluntary recall, underreporting, under-design, informed consent, risk communication, medical devices, FDA

## Abstract

Conflicting interpretations regarding the severity of the adverse effects associated with FDA-approved drugs and therapies are common among the United States Food and Drug Administration (FDA), the medical community, patients, and the general public. However, scholars have paid little attention to how these conflicting interpretations may affect the FDA’s reputation for facilitating inclusive dialogue between competing policy actors. Focusing on breast implants, a medical device characterized by a stormy regulatory past, we observe that the design properties of post-market surveillance are adjusted to low-quality information. Such information-gathering mechanisms likely lead to underreporting by medical practitioners and patients, thus resulting in low-quality data. Given that the FDA cannot rely on congressional appropriations to ensure a stable flow of funding, the confusion and uncertainty created by conflicting interpretations enhance the FDA’s ability to appeal to different audiences simultaneously and thereby secure funding from industry-based user fees. This strategy may persist until the FDA’s reputation is challenged by critical information regarding adverse effects and the ensuing potentially negative media coverage. A stable appropriation-based funding model will likely encourage stronger post-market surveillance of medical devices.

## 1 Introduction

Conflicting interpretations regarding the severity of the adverse effects associated with FDA-approved drugs and therapies are common among the United States Food and Drug Administration (FDA), the medical community, patients, and the general public. A case in point is the recent interaction between the American Association of Plastic Surgeons (AAPS) and the United States Food and Drug Administration (FDA) concerning the withdrawal of textured breast implants from the market to reduce the risk of an uncommon cancer. On February 27, 2024, an expert panel convened by the AAPS issued a statement, recommending that it may be “considered reasonable” to withdraw textured breast implants (1), while noting that the decision to do so “rests solely with the discretion of the surgeon in consultation with the patient” (2). In response, the FDA issued a statement on the following day, stating that “CDRH [Center for Devices and Radiological Health] welcomes thoughtful, scientific, and transparent public dialogue concerning breast implant safety and effectiveness […] [yet] [g]iven that the occurrence of [Breast Implant-Associated Anaplastic Large Cell Lymphoma] BIA-ALCL [Disease] is uncommon, prophylactic removal of textured implants is not recommended in asymptomatic patients” (3). Another example of conflicting interpretations is silicone breast implants and the risk of autoimmune/rheumatic disorders (4).

We analyze the sources of conflicting interpretations regarding breast implants and their potential impact on the FDA’s reputation for facilitating inclusive dialogue between competing policy actors. We draw on the concept of a regulatory error (5) and, more specifically, a repetitive regulatory error, defining the latter as a case in which at least two events either result from a regulatory error or are perceived as evidence of an earlier error from a scientific or media reputational perspective, even if this perception is erroneous. Such events may involve various temporal sequences of drug approval, withdrawal or moratorium, market re-approval or re-entry, and Black Box Warning (i.e., a warning that highlights severe or life-threatening risks).

We pose the following question: How does a reputation-sensitive regulator design its information gathering mechanisms in the case of a medical device with a safety profile that produced repetitive regulatory errors? To answer this, we focus on the FDA’s regulation of post-market surveillance in the case of breast implants, particularly how its reputation can withstand and even leverage conflicting interpretations and criticism from stakeholders (6). This specific case was selected for two reasons. First, although breast implants marketed before 1976 were exempt from demonstrating safety and effectiveness under an amendment to the Food, Drug, and Cosmetic Act—they were “grandfathered” into the market (7)—they have since been classified as high-risk medical devices, or Class III devices. Accordingly, they undergo a rigorous review process via the Premarket Approval (PMA) route, which is similar to the New Drug Application (NDA) review process for prescription drugs (8, 9). This rigorous process of scrutiny highlights the discrepancy between the use of breast implants for cosmetic purposes, on the one hand, and for medically necessary purposes, on the other. The fact that silicone breast implants are used for cosmetic reasons yet require long-term monitoring for implant-related symptoms and pathologies—along with the psychological distress associated with the risk of an uncommon cancer linked to textured implants—underscores the significant disparity between their perceived cosmetic benefits and potential adverse effects.

Second, certain breast implants have a stormy regulatory past (10, 11). Silicone breast implants were withdrawn from the market in 1992, their status was downgraded to investigational. The moratorium on the use of silicone gel-filled breast implants due to safety concerns ended in 2006, conditional on the implementation of post-approval studies by Allergan and Mentor. In 2015, Sientra voluntarily withdrew some implants from the United States market following an FDA warning, but they were reintroduced on March 1, 2016. Subsequently, in 2019, the FDA requested that Allergan issue a voluntary recall of its BIOCELL products (12). Allergan complied, leading to the removal of all textured Natrelle Breast Implants and Tissue Expanders from the global market. Unsurprisingly, “Breast implants are the most highly scrutinized devices approved by the US Food and Drug Administration” [(13), p. 212].

Indeed, as of October 2020, among major surgical devices, which include breast implants, arthroplasty implants, intravascular stents, nonabsorbable meshes, and pacemakers, only breast implant providers and facilities are required to comply with Patient Decision Checklist regulation (14). This in turn highlights the value of studying a singular medical device that differs so radically from others in terms of the reputational risk it poses to the FDA due to its tumultuous regulatory history. It also raises the expectation that it may be possible to establish robust post-market information-gathering tools to protect the public and, consequently, safeguard the FDA’s reputation.

Employing an information quality perspective, we analyze the core components of the post-market surveillance requirements employed in the case of breast implants, seeking to gauge whether current post-market surveillance regulation allows the FDA to capture sufficiently the diverse adverse effects of breast implants, based on which it can draw reliable conclusions. We therefore search for designs of information-gathering mechanisms that undermine the availability of quality information in the process of post-market surveillance (15). *Information* refers here to statistical and qualitative evidence as well as the beliefs that motivate professionals and mobilize the public (16).

## 2 An information quality perspective on post-market surveillance of breast implants

The Food and Drug Administration’s regulation does not end upon PMA. The FDA can require post-approval studies at the time of PMA approval. Regarding medical devices, the most common study type is the prospective cohort study (8, 426). As of 2021, according to the FDA, less than half (47%) of the 792 post-approval studies regarding medical devices ordered since 1991 were “completed,” with another 31% defined as having made “adequate” progress (8, 426). Regarding breast implants, even though the FDA ordered post-market surveillance studies of all major brands of breast implants in 2006, as of October 1, 2024, four are ongoing, two have been redesigned/replaced, four terminated, and one delayed (17). This lack of effective enforcement creates incentives for manufacturers to pay little attention to patients after implantation. Indeed, in 2011, the post-market studies of Allergan and Mentor Worldwide’s silicone gel-filled breast implants were in disarray. The manufacturers admitted that they had lost contact with many patients. Follow-up for Core Studies and Large Studies, which were conditions for approval, has been below target rates (18).

A lack of enforcement also leads to confusion among surgeons; indeed, each one can suggest a different estimation based on identified and de-identified data. Consequently, surgeons and patients find it difficult to reach credible decisions, which are especially necessary in cases of rare tumors that can sometimes be very aggressive. This problem is exacerbated by issues of underreporting, double-reporting, and lack of transparency. For example, in 2019, the FDA revealed that it had received over 300,000 adverse event reports concerning breast implants—more than 20 times the number previously disclosed to the public (19, p. 752). This situation was rectified, and all adverse event reports collected through alternative summary reporting were released in June 2019 (20).

Some patient registries exist, among them the National Breast Implant Registry, which is the product of FDA collaboration. This registry collects case reports by plastic surgeons concerning breast-implant-linked anaplastic large cell lymphoma (BIA-ALCL), which was found to be linked to textured implants. In addition, since 2022, it has gathered reports concerning squamous cell carcinoma, which is associated with all types of breast implants, and other lymphomas. However, participation in the registry is not mandatory and the registry does not include case reports detected by other physicians (21, 22), which, in this case, may refer to non-specialist plastic surgeons such as general surgeons; physicians without specialist surgical training who perform cosmetic surgery; and surgeons who undertake breast implant surgeries under the umbrella of cosmetic tourism. This, in turn, delays the issuing of public alerts by the FDA.

Patients have at their disposal two main sources of information: device labeling, especially the Black Box Warning, and an informed consent decision checklist that medical practitioners and facilities selling breast implants must review before surgery. With regard to the former, in 2021, the FDA issued a Black Box Warning to the breast implant manufacturers Allergan, Ideal Implant, Sientra, and Mentor (a subsidiary of Johnson & Johnson) covering information regarding adverse events, such as cancer and systemic symptoms (e.g., muscle pain, fatigue), which may develop over the lifespan of the device, and noting that the device’s lifespan is limited. Still, there is no requirement to include information in device labeling regarding the risks of squamous cell carcinoma. Rather, manufacturers are asked to do so. This may affect the timely awareness of patients and their response.

A similar problem arises with regard to the standardization and oversight of the informed consent process, the Patient Decision Checklist—introduced in October 2021—which is completed by all women contemplating breast implants. It includes mandatory topics to be discussed, requires the signatures of both patient and surgeon, and compliance with it allows the sale and distribution of breast implants to providers and facilities. The conversation with patients about to undergo breast implant surgery within the clinical space includes information regarding the rates of anaplastic large cell lymphoma linked to breast implants. However, no information is communicated regarding squamous cell carcinoma, and no accountability system is in place to monitor the quality of the informed consent process. This too may affect the timely awareness of patients and their response.

An additional important issue is voluntary device recalls, which “continue to be the fastest, most effective way for a company to correct or remove violative and potentially harmful products from the market” (23). However, the main communicative tool employed in the recall process is letters rather than electronic messaging. The use of traditional letters entails a potential delay in information dissemination, which may affect the timely awareness and response of both patients and healthcare providers. In addition, it may lead manufacturers to implement recall processes inconsistently.

Overall, the under-design of core components of post-market surveillance regarding breast implants leads to underreporting by medical practitioners and the public. Although not complex, these components produce conflicting interpretations due to low-quality data. Hospitals make bulk purchases of breast implants, and clinicians discuss costs and benefits based on this insufficient information. Efforts such as the National Evaluation System for Health Technology (NEST) aim to improve data collection by connecting clinical registries, electronic health records, and billing claims. However, NEST is a voluntary network that receives funding from both an FDA grant and the industry under the Medical Device User Fee Agreement. Although it uses so-called real-world evidence (24, 25), this cannot be considered a core pillar of the FDA’s information-gathering mechanisms because the evidence is largely drawn from healthcare claims data as opposed to objective clinical outcomes from randomized trials.

## 3 A reputational perspective on the US FDA’s post-market surveillance of breast implants

Conflicting interpretations regarding the severity of the adverse effects associated with FDA-approved breast implants may highlight the agency’s commitment to transparency and openness, its efforts to improve information quality, its capacity to adapt regulations, its promotion of stakeholder engagement, and its emphasis on balanced risk communication. With regard to the first, conflicting interpretations may indicate that the agency values transparency and welcomes open scientific debate. This may build trust with the public, demonstrating that the FDA is not hiding or manipulating data. Relatedly, acknowledging different viewpoints reinforces the FDA’s commitment to scientific integrity and the use of all available data, even if it is of varying quality.

Concerning the need for improved information quality, conflicting interpretations can emphasize the vital importance of higher quality data and well-honed research methodologies. The FDA can position itself as an advocate for improving standards and practices in medical device evaluations. A case in point is the FDA’s collaboration with medical device stakeholders to build the NEST as part of efforts to generate better evidence more efficiently across the total product lifecycle of medical devices for the purposes of device evaluation and regulatory decision-making. It can also justify the need for increased funding and support of more rigorous post-market surveillance, further reinforcing the FDA’s proactive stance on patient safety.

Concerning adaptive regulatory practices, by acknowledging conflicting interpretations the FDA can demonstrate that it is continuously updating and refining its regulatory practices based on new evidence and perspectives. A case in point is the 2022 FDA guidelines concerning the preparations companies should undertake to ensure rapid and effective voluntary product recall. This adaptability can enhance the FDA’s reputation as responsive to new information and dedicated to improving public health outcomes. Regarding stakeholder engagement, engaging with multiple stakeholders, including manufacturers, healthcare providers, and patients, to interpret and address the risks that medical devices entail can highlight the FDA’s commitment to collaborative problem-solving. This is especially applicable in the case of products with a troubled history, such as breast implants, urogynecologic surgical mesh, and others. With regard to the surgical mesh, the FDA indeed highlights its continued efforts to support women’s health and access to safe and effective medical devices. This is achieved by reviewing and analyzing published literature, Medical Device Reports (adverse event reports), and post-market information submitted to the FDA; conducting epidemiological research on the safety and effectiveness of surgical mesh; and collaborating with professional societies and other stakeholders to understand fully the post-market performance of relevant medical devices. Such an inclusive approach can strengthen the FDA’s reputation as a facilitator of comprehensive and diverse input in the decision-making process.

Regarding balanced risk communication, the FDA can use conflicting interpretations to communicate the complexities and uncertainties inherent to the risks that medical devices entail. This balanced risk communication can help manage public expectations and avoid alarmism. Balanced risk communication may help the FDA minimize public exposure to some of the criticism it receives by communicating more information on the relevant topic, framing this information optimally in ways that suit it, and encouraging conflicting interpretations. Balanced risk communication can also serve the FDA’s image as educating the public about the limitations and challenges of post-market surveillance data, fostering a more informed and understanding public, which can further support the FDA’s reputation.

In sum, conflicting interpretations regarding the severity of the adverse effects associated with FDA-approved breast implants derive from the under-design of information-gathering mechanisms but subsequently produce reputational dividends for the FDA (26). Yet, the flow of these dividends may cease when the consequences of the under-design of information-gathering mechanisms are publicly exposed. An example of this is the delay in issuing a cancer warning regarding Associated Squamous Cell Carcinoma, which resulted from delays and insufficient reporting of adverse events linked to breast implants. Thus, the idea that conflicting interpretations regarding the severity of the adverse effects associated with FDA-approved drugs and therapies are beneficial to the FDA has its limits. Unresolved conflicts over the interpretations of post-market surveillance as well as manufacturer accountability, regulatory jurisdiction, and other regulatory issues may undermine the FDA’s credibility in the eyes of some stakeholders. To manage reputational risk, the FDA tends to resolve conflicting interpretations by taking robust and swift regulatory action when new critical information emerges that could result in accusations of inaction or underreaction vis-à-vis the agency (27), particularly if this coincides with the potential for escalating negative media coverage.

When critical information emerges, it prompts the FDA to review its position and act swiftly and robustly to correct its (perceived) error. A case in point is the FDA’s jurisdictional claim vis-à-vis human tissue transplants involving corneal lenticules (a tissue product derived from the human cornea and applied to the cornea to correct vision problems). Between November 1989 and January 1990, five cases of AIDS infection, two of which involved corneal lenticules, were reported to the Center for Disease Control and Prevention (28). The FDA announced its jurisdictional claim over corneal lenticules in November 1989, without publicizing it in the *Federal Register* (29), perhaps in an effort to skip the 60-day public comments period. A similar process unfolded in the case of the FDA’s jurisdictional claim vis-à-vis dura mater (the outer meningeal tissue covering the brain, harvested from cadavers and used to patch the brain sacs of living patients). Likewise, this jurisdictional claim was not publicized in the *Federal Register* (29).

## 4 Looking ahead

Conflicting interpretations regarding the severity of the adverse effects associated with FDA-approved breast implants position the FDA as both too closely tied to industry and, at times, a guardian of public safety. Given that the FDA cannot rely on congressional appropriations to ensure a stable flow of funding, the confusion and uncertainty created by these conflicting interpretations enhance the FDA’s ability to appeal to different audiences simultaneously, thereby securing funding from industry-based user fees. In fact, reputational incentives encourage the FDA to continue with this strategy as long as this funding arrangement remains in place. Such a strategy may persist until the FDA’s reputation is challenged by critical information regarding adverse effects and the resulting potentially negative media coverage. A stable appropriation-based funding model is likely to alter the reputational incentives available to the FDA, leading a move to strengthen post-market surveillance of pharmaceutical drugs and devices.

In addition, with the increase in direct-to-consumer advertising, doctors, patients, and healthcare providers have come to be informed more by pharmaceutical advertising and less by clinical trial data, as well as the summary of that data on the device’s label. The outcome is an increasing array of conflicting interpretations highlighting how industry, the public, and medical providers are competing forces. To maintain its strong reputation in the face of these forces, the FDA primarily invests resources in solving the problems with the highest public visibility (30, 31) and the strongest industry demand: quick pre-market approval. Consequently, more resources are allocated to meeting the deadlines imposed by the User Fee Act, while weak post-market surveillance is masked by conflicting interpretations that justify either no action or delayed incremental action by the FDA.

This strategy, at the heart of which is the perpetuation of lower standards of post-market surveillance for medical devices, is not without risk. Devices that are approved quickly may entail safety problems; in turn this may elicit criticism of the FDA’s pre-market approval process, potentially undermining the FDA’s reputation for expertise. For this reason, public pressure should be exerted on the FDA to strengthen its post-market surveillance of breast implants, even requiring phase 4 trials to assess the safety of a device and how well it works in large, diverse populations over long periods. Following approval, device manufacturers should also be required to demonstrate patient outcome benefits in longer, and larger clinical trials.

## Data Availability

Publicly available datasets were analyzed in this study. This data can be found at: https://www.accessdata.fda.gov/scripts/cdrh/cfdocs/cfPMA/pma_pas.cfm.
